# Effect of Music Therapy on Anxiety, Stress and Sedative Requirements in Patients Undergoing Lower Limb Orthopedic Surgery Under Spinal Anesthesia: A Randomized Controlled Study

**DOI:** 10.7759/cureus.73809

**Published:** 2024-11-16

**Authors:** Usha Shukla, Urvashi Yadav, Thamarai K Kannan, Jay Brijesh Singh Yadav

**Affiliations:** 1 Anaesthesiology and Critical Care, Uttar Pradesh University of Medical Sciences, Saifai, IND; 2 Anaesthesiology, Uttar Pradesh University of Medical Sciences, Saifai, IND

**Keywords:** anxiety, music, orthopedic, sedation, spinal

## Abstract

Background

Music therapy is a safe, non-pharmacological way to help reduce anxiety, especially before surgery. It helps to calm the nervous system, promotes relaxation, and offers comfort by blocking outside noise and distracting from pain. This can be helpful in managing preoperative anxiety and symptoms like hypertension and tachycardia. While the benefits of music therapy for stress and sedation are well known, its specific effects on patients receiving spinal anesthesia haven't been studied yet. Understanding this could improve care for these patients. Overall, music therapy could be a valuable tool in the surgical process.

Objectives

*Primary objective*: The study aimed to compare sedative requirements between two patient groups.

*Secondary objectives*: To compare anxiety levels between two patient groups using the State-Trait Anxiety Inventory. It also assessed hemodynamic changes and measured stress levels by analyzing serum cortisol and immunoglobulin A levels in both groups.

Materials and methods

Ninety-two American Society of Anesthesiologists physical status class I and II patients, aged 18-65 years, with a body mass index of 18-25 kg/m^2 ^undergoing lower limb orthopedic surgery under spinal anesthesia were selected for the study. After spinal anesthesia, the group M (n=46) patients received the music of their choice from headphones connected to mobile phones and patients in group NM (n=46) were attached headphones without any music therapy. After the attachment of headphones, propofol was administered in both the groups for sedation in a bolus dose of 1-2mg/kg iv followed by an infusion dose of 5-50 μg/kg/min. The propofol infusion was titrated based on Bispectral Index (BIS) values kept between 70 and 80 for moderate sedation. Preoperatively, blood samples were collected to measure baseline serum cortisol and IgA levels. Intraoperatively, hemodynamic parameters were measured, and anxiety level was assessed using the State-Trait Anxiety Inventory (STAI) Scale 30 minutes prior to the administration of spinal anesthesia. Postoperatively, anxiety was re-evaluated, and additional blood samples for the assessment of cortisol and IgA were taken at 30 minutes and 12 hours after the administration of spinal anesthesia.

Results

The mean serum cortisol was lower in Group M as compared to Group NM (15.1±1.2 vs. 17.1±1; p = 0.0001). Mean serum IgA was significantly lower in Group M as compared to Group NM (269.3±54.5 vs. 294.2±49.9; p = 0.024) during the intraoperative period. The mean STAI Score was lower in Group M compared to Group NM (34.87±4.53 vs. 34.61±5.06; p = 0.008). The mean propofol requirement (mg) was lower in Group M as compared to Group NM (147.8±11.3 vs. 193±16; p = 0.0001). The hemodynamic parameters were comparable between the groups (p>0.05).

Conclusion

Patients in the music therapy group experienced lower anxiety, stress, and serum cortisol levels during surgery, with reduced serum IgA levels and decreased propofol requirement for sedation. Overall, music therapy was effective in reducing anxiety, stress, and sedative requirements during surgery under regional anesthesia.

## Introduction

Spinal anesthesia is frequently used in orthopedic surgeries due to its benefits, including effective pain control, stable vital signs, quicker recovery, and reduced risks compared to general anesthesia [1]. However, the operating room can be very noisy, particularly with the use of hammers, drills, and oscillating saws, which can reach noise levels up to 105 decibels [2]. This noise, coupled with heightened awareness during spinal anesthesia, may increase patient anxiety and the need for additional sedatives [3]. Such anxiety can lead to greater pharmaceutical use, potential procedure cancellations, and decreased patient satisfaction [4].

Music therapy offers a non-invasive, drug-free solution for managing anxiety in surgical settings [5,6]. It helps by reducing sympathetic nervous activity and activating the parasympathetic nervous system, leading to decreased anxiety and enhanced relaxation [7]. By providing emotional comfort, masking external noises, and serving as a distraction, music therapy can mitigate preoperative anxiety and help manage symptoms like high blood pressure and elevated heart rate [8]. Studies showed that music played through headphones can further decrease anxiety and improve overall patient satisfaction [9].

Surgery triggers stress responses that affect both hormonal and immune systems. For instance, cortisol, a stress hormone, increases in response to both physiological and psychological stress, impacting various bodily functions [10]. Additionally, stress can cause short-term increases in immunoglobulin A (IgA), an antibody important for immune function [11]. Although music therapy has been shown to help with anxiety in many surgical settings, there is limited research on its effects in patients receiving spinal anesthesia. This has led to a planned study to explore how music therapy can affect anxiety, stress, and the need for sedatives during elective lower limb orthopedic surgeries with spinal anesthesia.

## Materials and methods

This prospective randomized controlled study was conducted after obtaining the approval from institutional ethical committee (42/2022-23) dated December 22, 2022. The study was registered in the clinical trial registry of India (CTRI/2024/05/067076). The study was conducted between May 15, 2024 and October 15, 2024. After obtaining informed consent from the patients, 92 individuals classified as American Society of Anesthesiologists Physical Status class I and II patients, aged 18-65 years, with a body mass index of 18-25 kg/m², were enrolled in the study to undergo lower limb orthopedic surgery under spinal anesthesia. Patients who refused to listen to music or had a history of head and neck surgeries, psychiatric disorders, or hearing defects were excluded from the study.

Sample size calculation and randomization

Taking into consideration an alpha error of 0.05 and power of study as 95%, the projected sample size was revealed to be 46 patients per group. The following sample size equation was used [12]:

$\ n = \frac{(Z_{\alpha/2} + Z_{\beta})^2 (\sigma_1^2 + \sigma_2^2)}{(\mu_1 - \mu_2)^2}$

Here, *n* = sample size per group, $Z_{\alpha/2}$ = Z-score corresponding to the desired confidence level (e.g., 1.96 for a 95% confidence level), $Z_{\beta}$=Z-Score corresponding to the desired power (e.g., 0.84 for 80% power), $\sigma_1^2$ = Variance of the treatment group is 2.07, $\sigma_2^2$​ = Variance of the control group is 5.52, μ_1_−μ_2_= Minimum clinically significant difference between the two means.

Randomization was done using computer-generated random number table and the group allocation was done through sequentially numbered sealed opaque envelope technique. A total of 92 patients were assigned to two groups of 46 patients each.

Group M (n=46) patients received the music of their choice (collection of 20 light soothing music tracks) from headphones connected to mobile phones after administration of spinal anesthesia and continued till the end of surgery.

Group NM (n=46) patients did not receive any music. Headphones were attached for blinding purposes.

In the preoperative room, under all aseptic precautions, peripheral vascular access was obtained with 18-gauge intravenous cannula in all the patients. Under all aseptic precautions, the blood sample was drawn and sent to the laboratory to measure the basal level of the serum cortisol and the serum IgA level. Thereafter, ringer lactate infusion was started at the rate of 10-15 ml/kg body weight.

In the operating room, the American Society of Anesthesiologists (ASA) standard monitoring devices, non-invasive blood pressure, electrocardiography, and pulse oximetry were attached and the baseline parameters were recorded. The Mindray BIS module (Mindray, Shenzhen, China) with four sensor electrodes were attached to the forehead of the patients. The electrodes were placed on the following areas: Electrode 1, center of forehead, about two inches above the bridge of the nose; Electrode 2, directly over the patient's eyebrow; Electrode 3, temple, between the outer corner of the eye and the hairline, and Electrode 4, flat over the outer half of the eyebrow. The Bispectral Index (BIS) reading was taken as baseline parameters.

The spinal anesthesia was administered under all aseptic precautions with 3-3.5 ml, 0.5% hyperbaric bupivacaine in L4-L5 intervertebral space with 25G Quincke’s spinal needle in a sitting position. After adequate sensory and motor levels were achieved, patients were given intervention as per the assigned group. Propofol was administered in both groups for sedation in a bolus dose of 1-2mg/kg iv followed by an infusion dose of 5-50 μg/kg/min. The propofol infusion was titrated based on BIS values between 70 and 80 for moderate sedation and maintaining the blood pressure within 20% of baseline. Propofol infusion was stopped if the fall in blood pressure was 20% of the baseline and the i.v. (intravenous) fluid was increased accordingly. Monitoring of vitals was done by anesthetists who were blinded to an intervention used in each group.

Data collection

All the patients were monitored for hemodynamic parameters like heart rate, blood pressure, and oxygen saturation at baseline, 5 minutes, 10 minutes, 15 minutes, 20 minutes, 30 minutes, 40 minutes, 1 hour and 2 hours till the completion of the surgery. The total consumption of propofol was calculated with the target BIS value of 70-80.

The patient’s anxiety level was assessed using the State-Trait Anxiety Inventory (STAI) Scale in the preoperative room at 30 minutes before spinal anesthesia and at 30 minutes after the completion of the surgery. In STAI has 20-item self-report assessment [13] (Table 1).

**Table 1:** State-Trait Anxiety Inventory (STAI) Scale [13]

	STAI	Not at all	A little	Somewhat	Very Much So
1	I feel calm	1	2	3	4
2	I feel secure	1	2	3	4
3	I feel tense	1	2	3	4
4	I feel strained	1	2	3	4
5	I feel at ease	1	2	3	4
6	I feel upset	1	2	3	4
7	I am presently worrying over possible misfortunes	1	2	3	4
8	I feel satisfied	1	2	3	4
9	I feel frightened	1	2	3	4
10	I feel uncomfortable	1	2	3	4
11	I feel self-confident	1	2	3	4
12	I feel nervous	1	2	3	4
13	I feel jittery	1	2	3	4
14	I feel indecisive	1	2	3	4
15	I am relaxed	1	2	3	4
16	I feel content	1	2	3	4
17	I am worried	1	2	3	4
18	I feel confused	1	2	3	4
19	I feel steady	1	2	3	4
20	I feel pleasant	1	2	3	4

A score of 20-37 was considered low anxiety, 38-44 was considered moderate anxiety, and 45-80 was considered high anxiety. The blood sample for the serum cortisol and the serum IgA was drawn at 30 minutes and 12 hours after the spinal anesthesia.

Bradycardia was treated with incremental doses of injection atropine 0.3mg intravenous. Desaturation was treated with moist O_2_ inhalation at 5 litre/min. Hypotension was managed with a bolus of crystalloids or with increments of injection mephentermine 6mg i.v. as required.

Statistical analysis

Descriptive analysis of quantitative parameters was expressed as mean and standard deviation. Categorical data were expressed as the absolute number and percentage. Shapiro-Wilk test was used for the testing of normality assumption of quantitative data. Independent Student's t-test was used for testing of mean between independent groups. Paired t-test was used for testing of mean in the same groups. Cross tables were generated and chi-square test was used for testing of associations. A P-value < 0.05 was considered statistically significant. The analysis was done using IBM SPSS Statistics, version 25.0 (IBM Corp., Armonk, NY).

## Results

**Figure 1:**
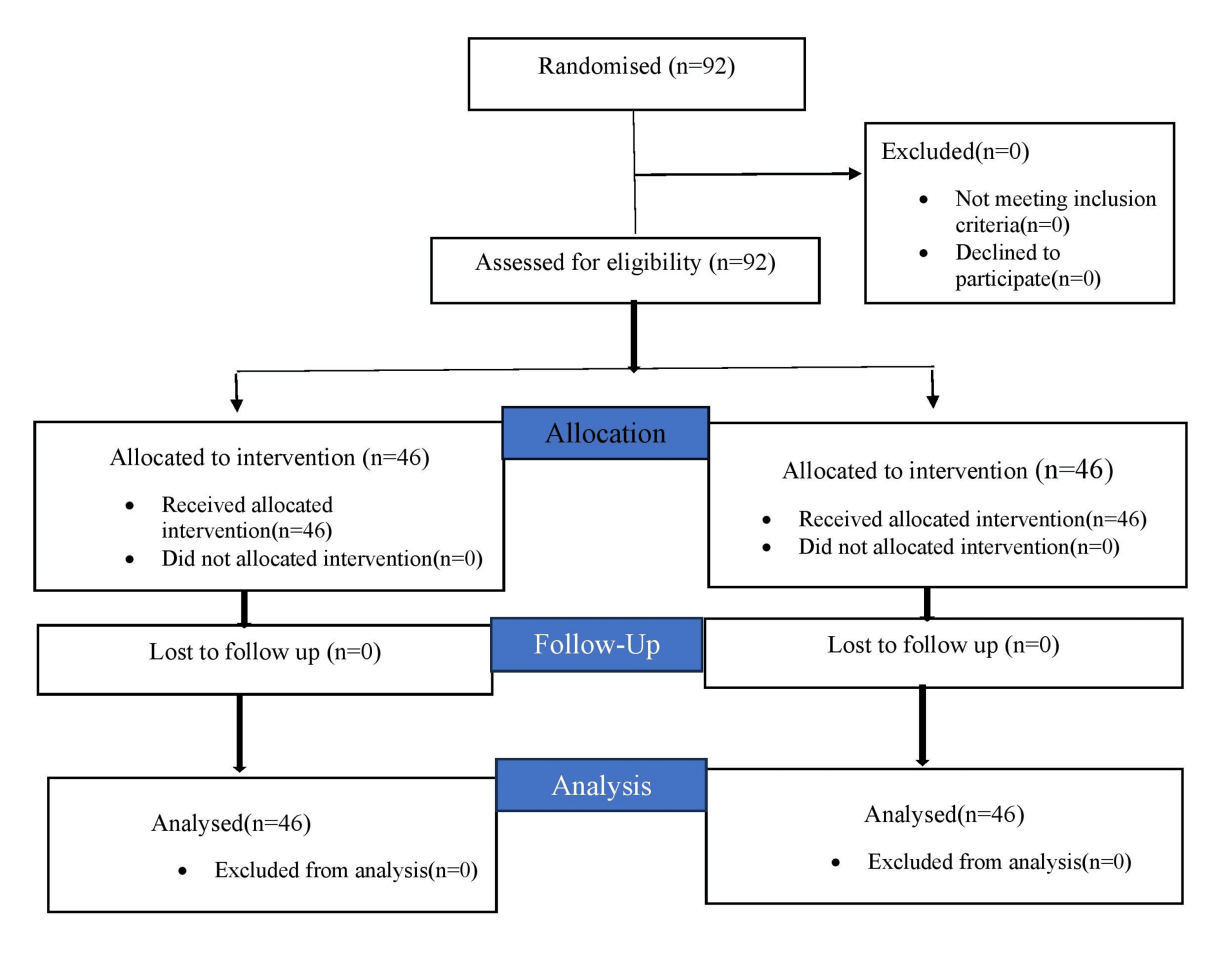
CONSORT (Consolidated Standards of Reporting Trails) Diagram

None of the patients were excluded from our study (Figure 1). A total of 92 patients were enrolled in the study divided into two groups of 46 each.

Table 2 shows that demographic parameters were comparable among both groups.

**Table 2:** Demographic characteristics of the groups Group M: music, Group NM: non-music, ASA PS class: American Society of Anesthesiologists Physical Status class, BMI: body mass index. P>0.05, statistically not significant; superscript 1: unpaired t-test; superscript 2: chi-square test.

Characteristics		Group M (n=46) (Mean±SD)	Group NM (n=46) (Mean±SD)	P value
Age (years)		37.8±12.1	33.2±10.2	0.098^1^
BMI (kg/m^2^)		24.0±0.9	23.8±1.0	0.227^1^
ASA Physical Status classification	I	2.2%	2.2%	1.000^2^
	II	97.8%	97.8%	
Duration of surgery (minutes)		151.19±18.18	149.72±17.98	0.6851^1^

**Figure 2:**
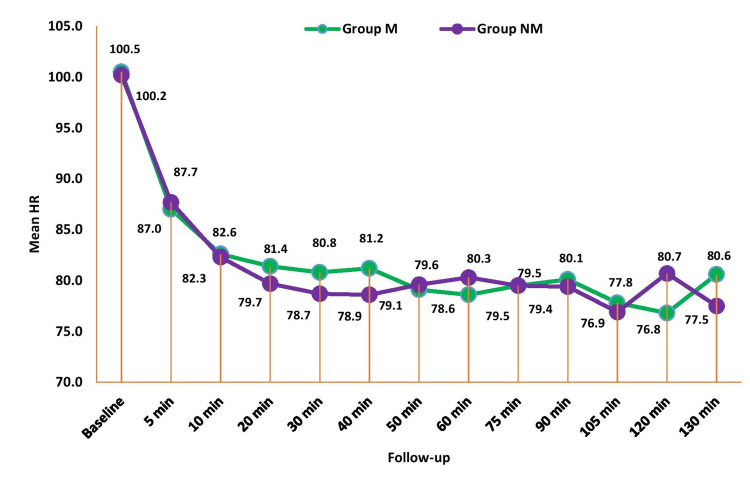
Comparison of heart rate (beats/min) between the study groups

Figure 2 shows the comparison of heart rate (HR) between the groups across periods. There was no significant mean difference in HR between the groups at all the time periods (p>0.05).

**Figure 3:**
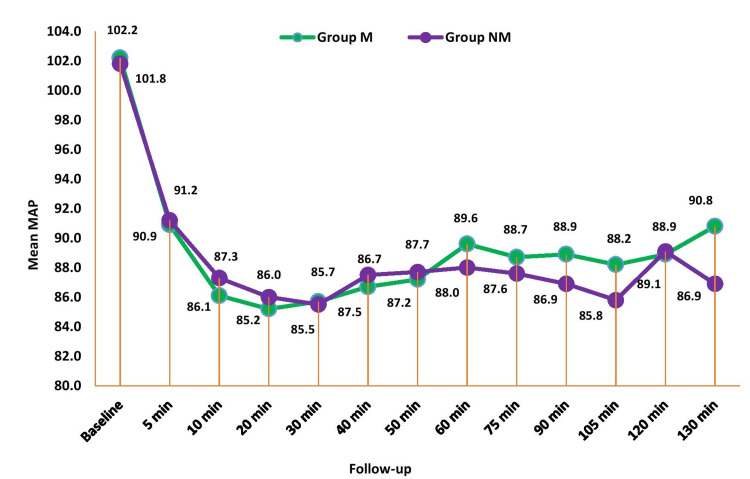
Comparison of mean arterial pressure (mmHg) between the study groups

Figure 3 shows the comparison of mean arterial pressure (MAP) between the groups across periods. There was no significant mean difference in MAP between the groups at all the time periods (p>0.05). There was no significant mean difference in SpO_2_(oxygen saturation) between the groups at all time periods (p>0.05).

**Figure 4:**
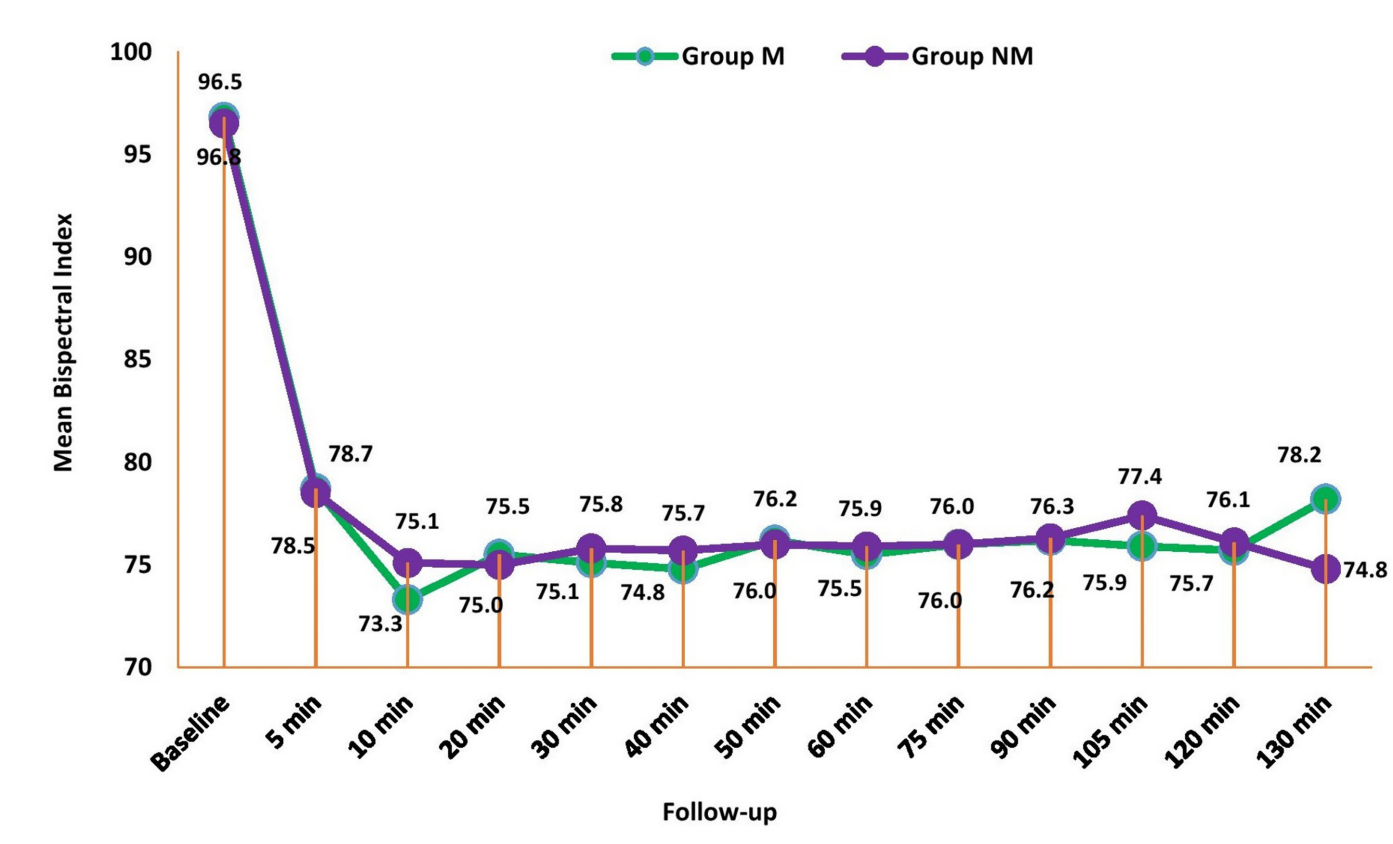
Comparison of Bispectral Index (BIS) between the study groups

Figure 4 shows the comparison of BIS index between the groups across periods. There was no significant mean difference in BIS index between the groups at all the time periods (p>0.05).

Table 3 shows the comparison of STAI scores between the study groups during the preoperative and postoperative periods.

**Table 3:** Comparison of STAI Score between the study groups during preoperative and postoperative periods STAI: State-Trait Anxiety Inventory; Group M: music; Group NM: non-music; SD: standard deviation, *p<0.05, statistically significant; superscript 1: unpaired t-test.

STAI Score	Group M (n=46), Mean± SD	Group NM (n=46), Mean± SD	t-value	P-value
Preoperative	55.28±5.91	55.57±4.92	-.249	0.804^1^
Postoperative	34.87±4.53	37.6±5.06	-2.735	0.008^1 ^*
t-value	17.655^1^	16.947		
P-value	0.007^1^*	0.009^1^*		

*Intergroup comparison: *In the preoperative period, Group M had a lower mean STAI score (55.28 ± 5.91) compared to Group NM (55.57 ± 4.92), but the mean difference was statistically not significant between the groups (p=0.804). Postoperatively, Group M had a lower mean STAI score (34.87 ± 4.53) compared to Group NM (37.61 ± 5.06), but the mean difference was statistically not significant between the groups (p=0.008).

*Intragroup comparison*: In group M, the mean STAI Score during the preoperative and postoperative periods was 55.28 ± 5.91 and 34.87±4.53, and this mean difference was statistically significant between the groups (p= 0.007). In group NM, the mean STAI Score during the preoperative and postoperative periods was 55.57 ± 4.92 and 37.61 ± 5.06; this mean difference was statistically significant between the groups (p=009).

Table 4 shows the comparison of serum cortisol (mg/dl) and serum IgA level(µg/dl) between the study groups during preoperative, intraoperative, and postoperative periods.

**Table 4:** Comparison of serum cortisol (mg/dl) and serum IgA(µg/dl) level between the study groups Group M: Music; Group NM: non-music; SD: standard deviation; SE: standard error of mean; CI: confidence interval; *p<0.05, statistically significant; superscript 1: unpaired t-test.

Serum Immunoglobulin A	Group M (n=46), Mean ± SD	Group NM (n=46), Mean ± SD	Mean Difference ± SE	95% CI of the Difference		t-value	*p- value
				Lower	Upper		
Preoperative	450.6±30.8	451.6±30.8	-0.5±6.2	-12.8	11.9	-0.074	0.941^1^
Intraoperative	269.3±54.5	294.2±49.9	-24.9±10.9	-46.6	-3.3	-2.291	0.024^1^*
Postoperative	358.4±45.7	354.7±45.1	3.7±9.5	-15.1	22.5	0.390	0.698^1^
Serum Cortisol							
Preoperative	24.8±3.5	23.6±3.1	1.2±0.7	-0.2	2.6	1.756	0.082^1^
Intraoperative	15.1±1.2	17.1±1	-1.9±0.2	-2.4	-1.5	-8.913	0.0001^1^*
Postoperative	18.6±1.5	18.3±1.3	0.4±0.3	-0.2	0.9	1.275	0.205^1^

In the preoperative period, Group M had a mean serum IgA level of 450.6±30.8 µg/dl, lower than Group NM's level (451.6±30.8 µg/dl), and the mean difference was statistically not significant between the groups (p=0.0941). During the intraoperative period, the mean serum IgA level was significantly lower in Group M (269.3±54.5 µg/dl) compared to Group NM (294.2±49.9 µg/dl) and there was a statistically significant difference between the groups (p=0.024). During the postoperative period, Group M had a mean serum IgA level of 358.4±45.7 µg/dl, higher than Group NM's value (354.7±45.1 µg/dl), and the mean difference was statistically not significant between the groups (p=0.698).

In the preoperative period, Group M had a higher mean serum cortisol level (24.8±3.5 mg/dl) compared to Group NM (23.6±3.1 mg/dl), and the mean difference was statistically not significant between the groups (p=0.082). During the intraoperative period, Group NM had a significantly higher mean serum cortisol level (17.1±1 mg/dl) than Group M (15.1±1.2 mg/dl), and the mean statistically significant difference between the groups (p=0.0001). Postoperatively, serum cortisol levels were similar between the groups, with Group M at 18.6±1.5 mg/dl and Group NM at 18.3±1.3 mg/dl, and this mean difference was statistically not significant between the groups (p=0.205).

Table 5 shows the comparison of propofol consumption (mg) during surgery between the study groups.

**Table 5:** Comparison of Propofol consumption (mg) during surgery between the study groups Group M: Music; Group NM: non-music; SD: standard deviation; SE: standard error; CI: confidence interval; *p<0.05, statistically significant; superscript 1: unpaired t-test.

	Group M (n=46) Mean ± SD	Group NM (n=46) Mean ± SD	Mean Difference ± SE	95% CI of the Difference		t-value	P-value
				Lower	Upper		
Propofol consumption during surgery (mg)	147.8±11.3	193±16	-45.2±2.9	-51.0	-39.5	-15.616	0.0001^1^*

The mean propofol consumption was lower in Group M (147.8±11.3 mg) compared to Group NM (193±16 mg) and the mean difference was statistically significant between the groups (p=0.0001).

## Discussion

Intraoperative anxiety can be managed with large doses of sedatives, but these may impair circulation and respiration, making non-pharmacologic methods attractive [14]. Music therapy, a non-drug intervention, has demonstrated benefits in reducing anxiety and blood pressure during awake surgeries [15] and improving pain scores and satisfaction under general anesthesia [16]. It is a cost-effective, low-risk approach that can decrease surgical stress and improve patient experience and satisfaction, particularly in lower limb orthopedic surgeries [17]. In our study, the demographic profile of patients like age, ASA PS Class, and BMI were comparable between the groups (p>0.05). Hemodynamic parameters like heart rate, mean arterial pressure, and oxygen saturation were recorded in both groups and the mean values were comparable in terms of hemodynamic parameters at all time periods of the study (p>0.05).

State Trait Anxiety Inventory (STAI) score

In our study, during the preoperative period, no significant difference in mean STAI scores was observed (p>0.05) between groups. Group M had a lower mean STAI score than Group NM (34.87±4.53 vs. 37.61±5.06; p=0.008) during the postoperative period. This reduction in anxiety may be attributed to the activation of the limbic system and auditory pathways, which interact with the reticular activating system, hippocampus, and hypothalamus, leading to decreased excitatory neurotransmitters and promoting relaxation. Supporting our findings, Kukreja et al. [12] reported a lower postoperative STAI score in Group M (28.14±1.0) versus control (34.71±2.31; P = 0.01)]. Similarly, Zengin et al. [18] found lower scores in the MI group (38.74±8.94) compared to controls (43.26±6.92; P = 0.006). Labrague et al. [19] also observed reduced STAI scores in the experimental group (36.43±1.86) versus controls (43.30±2.02; P<0.05), aligning with our results.

Serum cortisol

During stress, cortisol is released by the adrenal glands as part of the body's "fight or flight" response. It helps regulate energy by increasing glucose availability, suppressing non-essential functions, and enhancing brain function for quick decision-making. Prolonged elevated cortisol levels, however, can lead to negative effects such as immune suppression, anxiety, and cognitive impairments [20,21]. In the present study, there was no significant difference in mean serum cortisol levels between groups during the preoperative and postoperative periods (p > 0.05). However, intraoperatively, Group M showed a significantly lower mean serum cortisol level than Group NM (15.1 ± 1.2 mg/dl vs. 17.1 ± 1 mg/dl; p = 0.0001), suggesting the potential of music therapy to reduce stress through hypothalamic-pituitary-adrenal (HPA) axis modulation. Graversen and Sommer [22] similarly found lower cortisol levels in a Soft Music group during laparoscopic cholecystectomy (p<0.05). Consistently, Zengin et al. [18] reported lower levels in the music intervention group (14.82±4.16) compared to controls (16.63±2.81; p=0.012). Likewise, Koelsch et al. [3] observed reduced cortisol in a music group undergoing elective total hip replacement (p<0.05), reinforcing our findings.

Serum immunoglobulin A

Acute stress can temporarily increase serum IgA levels, potentially enhancing mucosal immunity to address immediate threats. This increase is part of the body’s adaptive response to stress. However, prolonged stress typically leads to a decrease in IgA levels. Chronic stress can ultimately weaken immune defences over time [23,24]. In our study, no significant differences were observed in mean serum IgA levels among the groups pre-operatively and post-operatively (p > 0.05). However, intraoperatively, Group M had a lower mean serum IgA level (269.3 ± 54.5 µg/dl) compared to Group NM (294.2 ± 49.9 µg/dl), which was statistically significant (p=0.024). This reduction may indicate that music lowers serum IgA levels, reflecting an acute stress response influenced by immune activity. Nilsson et al. [11] conducted a randomized controlled study with 75 patients under general anaesthesia, divided into three groups. They found no significant differences in mean serum IgA levels across the groups (p>0.05), likely due to the uniform anaesthesia type administered to all participants.

Propofol consumption during surgery

In our study, we aimed to maintain BIS values between 70 and 80 by titrating propofol doses (5-50 µg/kg/min). We found a statistically significant difference in propofol consumption between groups: group M propofol consumption was 147.8±11.3 mg, while group NM it was 193±16 mg (p=0.0001). Limited studies exist on propofol consumption under regional anaesthesia, but Koelsch et al. [3] reported lower propofol use in a music group compared to controls (p<0.05).

Limitations of the study

One of the drawbacks of this study is the high cost of investigations associated with measuring serum cortisol and serum immunoglobulin A levels. These tests require specialized equipment and resources, making them expensive to perform, which may limit their accessibility and feasibility within the study. The potential additive or synergistic effects of combining music therapy with propofol require further investigation and present an intriguing area for future research.

## Conclusions

In the postoperative period, the mean STAI score of group M was significantly lower than that of the non-music group, indicating reduced anxiety. The mean serum cortisol levels of the music group during the intraoperative period were noticeably lower, suggesting decreased stress. Serum IgA levels were also lower in the music group, further reflecting reduced stress levels. Furthermore, the music group required less propofol on average compared to the non-music group, indicating lower sedative needs. Overall, these findings suggest that music therapy significantly alleviates anxiety, stress, and sedative requirements in patients undergoing surgery under regional anesthesia.
